# Migraine as a risk factor for primary open angle glaucoma: A systematic review and meta-analysis

**DOI:** 10.1097/MD.0000000000011377

**Published:** 2018-07-13

**Authors:** Chang Xu, Jingjing Li, Zhi Li, Xiaochun Mao

**Affiliations:** Department of Ophthalmology, Xiangyang Central Hospital, Xiangyang, Hubei, P.R. China.

**Keywords:** meta-analysis, migraine, primary open angle glaucoma

## Abstract

Migraine is increasingly being reported as a risk factor for primary open angle glaucoma (POAG). However, studies aimed to investigate this association yielded conflicting results. To assess the consistency of the data on the topic, we performed a systematic review and meta-analysis. A systematic literature search from Embase, Web of Science, and PubMed was performed to identify relevant studies on the relationship between migraine and POAG. Random effects models were used to estimate the pooled relative risks (RRs) with 95% confidence intervals (95% CIs) in this meta-analysis. A total of 11 studies meeting the inclusion criteria were included in this meta-analysis. Our findings showed an RR of developing POAG of 1.24 (95% CI = 1.12–1.37) in migraine patients. No evidence of significant heterogeneity was detected across studies (*P* = .071; *I*^2^ = 41.7%). This association was not modified by the glaucoma type of the included patients. A significant association was observed in case–control design studies, but not in cohort design studies. Little evidence of publication bias was found. The findings of this meta-analysis suggest that migraine can significantly increase the risk of the development of POAG. However, the cohort study design failed to identify this association. Whether migraines can significantly increase the risk of developing POAG is still controversial.

## 1 Introduction

Glaucoma is a multifactorial condition characterized by a progressive optic neuropathy and distinctive visual field loss and has become the most common cause of irreversible blindness worldwide.^[1,2]^ The exact mechanism by which anatomic and functional damage is inflicted on patients with primary open angle glaucoma (POAG) remains unknown. The main risk factor for POAG is old age,^[3]^ with an increasing risk for POAG of 1.73 for each decade increase in age over 40 years.^[2]^ In addition, ethnic background (especially African),^[4]^ family history of glaucoma, elevated intraocular pressure (IOP), and high myopia are all known risk factors for POAG.^[5–8]^

Recent epidemiologic studies have suggested that migraine may be associated with POAG, although these findings have been inconclusive and conflicting. For example, Lin et al^[9]^ found that subjects with migraine were 1.2 times more likely to have POAG compared with those without migraine, even after adjustment for the risk factors such as gender, age, monthly income, and level of urbanization of the community. However, another study on Chinese cohorts reported that migraines did not increase the risk of POAG.^[10]^

To date, the pathogenesis of POAG is not totally understood. A clearer understanding of the association between migraine and POAG may therefore provide insights into the pathophysiology of this disease. For this reason, we conducted this meta-analysis of the available published literature to examine the potential relationship between migraine and POAG. The major drawback of cross-sectional studies is that they cannot establish a clear, temporal relationship between exposure and outcome. Thus, only case–control and cohort studies were included in this meta-analysis.

## 2 Patients and methods

### 2.1 Search strategy

The study was performed according to the recommendation of the Preferred Reporting Items for Systematic Reviews and Meta-analysis (PRISMA) guidelines.^[11]^ A systematic literature search from Embase, Web of Science, and PubMed was performed to identify relevant studies on the relationship between migraine and POAG published up to October 2017. The following search terms were used: glaucoma, IOP, ocular hypertension, intraocular hypertension, migraine, cephalagra, and hemicrania. Additional information was obtained by searching Google Scholar. We also screened the reference lists of all retrieved trials to identify studies not yet included in the computerized databases. The search did not restrict the language, methodological filter, or publication year.

### 2.2 Inclusion and exclusion criteria

The following inclusion criteria were met in the present meta-analysis: the study design was a cohort or case–control design; the study evaluated the association between migraine and POAG; ORs or relative risks (RRs) estimates with their 95% confidence intervals (CIs) were provided (or sufficient data were provided to calculate ORs or RRs values). The following exclusion criteria were also considered: studies focused on angle-closure glaucoma or secondary glaucoma rather than POAG; crude data did not provide RRs or ORs or sufficient data for their calculation; the reports were letters, reviews, case reports, or abstracts, or reports with incomplete data. If multiple publications from the same study population were available, then duplicate analyses were checked and only the most recent publication was included.

### 2.3 Data extraction and quality assessment

The following information was extracted by 2 independent reviewers: publication year, first author, study design, the ascertainment method of migraines, definition of glaucoma, age of subjects, sample size, the provided adjusted ORs or RRs with their 95% CIs or the data for calculating the ORs, the adjusted variables, and the methods for selecting study participants. The study quality was assessed by 2 reviewers using the tool described by Sanderson et al.^[12]^ The variables of the methods used for selecting study subjects, the methods used for measuring outcomes and exposure, the methods used to control for confounding, design-specific sources of bias, potential conflicts of interest, and statistical methods were examined.

### 2.4 Statistical analyses

We conducted this meta-analysis using the Stata software package (Version 12.0; Stata Corp., College Station, TX). We assessed the correlation between migraine and POAG by estimating the pooled RR with 95% CI using the random-effects model. Using the rare disease assumption, RR in cohort studies and the OR in case–control studies were integrated to estimate the pooled RR.^[13,14]^ We evaluated the presence of among-studies heterogeneity using the *χ*^2^ and *I*^2^ tests. For the *χ*^2^ test, *P* < .05 was considered to represent significant heterogeneity. For *I*^2^, a value >50% indicated significant heterogeneity.^[15]^ We conducted a stratified analysis based on the study's design (case–control, nested case–control/cohort study), the methods used to determine migraines (medical records, self-reports), the geographical area (North America, Europe, Asia, and Australia), the type of glaucoma (POAG, normal tension glaucoma/POAG (studies only included the POAG patients were defined POAG subgroup; studies included both POAG and NTG patients were defined NTG/POAG subgroup)), and the adjusted variables for age and sex, diabetes, and hypertension. The reliability of the outcomes of the meta-analysis was determined by a sensitivity analysis performed by omitting each individual study one at a time. Finally, publication biases were detected using the Begg and Egger tests and assessed using Begg funnel plots.^[16,17]^*P* < .05 was considered statistically significant in the test results of overall effect.

## 3 Results

### 3.1 Literature search

**Figure 1:**
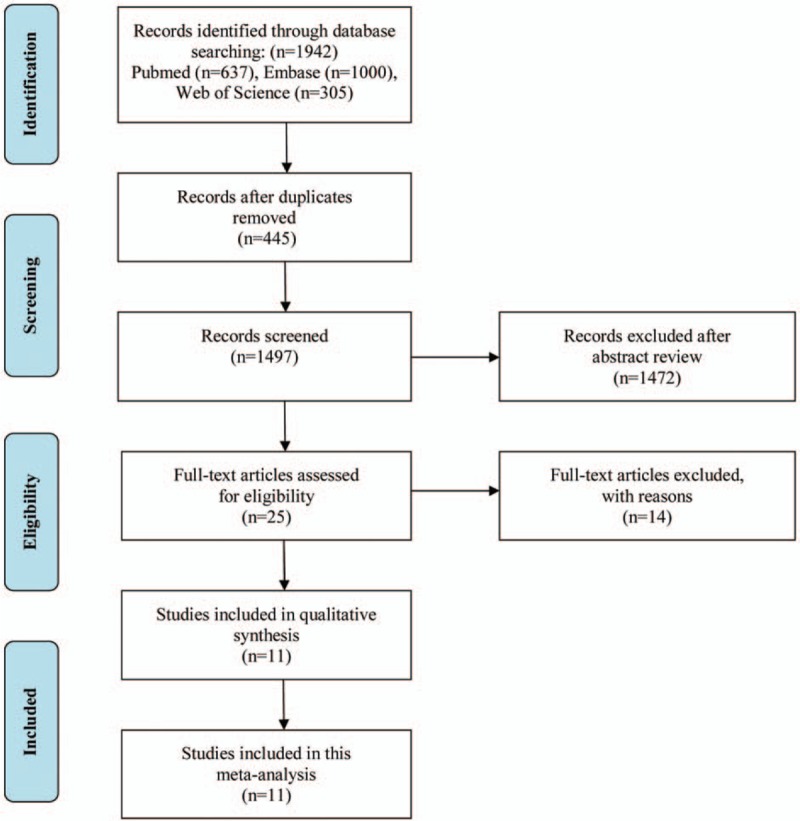
PRISMA flow diagram showing study selection.

A total of 1942 papers were identified through literature searches of 3 databases. Of these, 445 were duplicate publications and were removed. A further 1472 papers were also excluded following title and abstract review. Of the remaining 25 publications retained for further assessment and a full-text review, nine papers were excluded for the following reasons: review (n = 1)^[18]^; no focus on the relationship between the migraine and POAG (n = 3)^[19–21]^; comparison of the incidence of migraine between familial and sporadic glaucoma (n = 1)^[22]^; no provision of RRs or ORs or data for their calculation (n = 3)^[23–25]^; letter (n = 1)^[26]^; not case–control or cohort design (n = 5).^[27–31]^ Ultimately, 11 studies,^[9,10,32–40]^ including 3 cohort or nested case–control,^[10,32,35]^ and 8 case–control studies,^[9,33,34,36–40]^ were included in the present meta-analysis. The detailed process of data selection is described in Fig. 1.

### 3.2 Characteristics of studies and quality assessment

Table 1 displays the characteristics of the included studies. These studies were performed in different locations, including Canada, the United States, Japan, Australia, Portugal, Denmark, Germany, and Chinese Taiwan, and were published between 1975 and 2016. The sample sizes in the included studies ranged from 148 to 306,692. Some of the studies included only POAG patients and others included both POAG and normal tension glaucoma (NTG) patients. The methods used to determine migraine varied across studies. Four studies ascertained the diagnosis of migraine by self-reports^[37–40]^ and 7 by medical records.^[9,10,32–36]^ The methods used to determine glaucoma varied across the studies. Most studies defined POAG based on glaucomatous visual field loss and glaucomatous optic neuropathy. Several studies included other additional factors, such as open angle, elevated IOP, and the exclusion of angle closure or secondary glaucoma. A detailed quality assessment of all the included studies is displayed in Table 2.

**Table 1:**
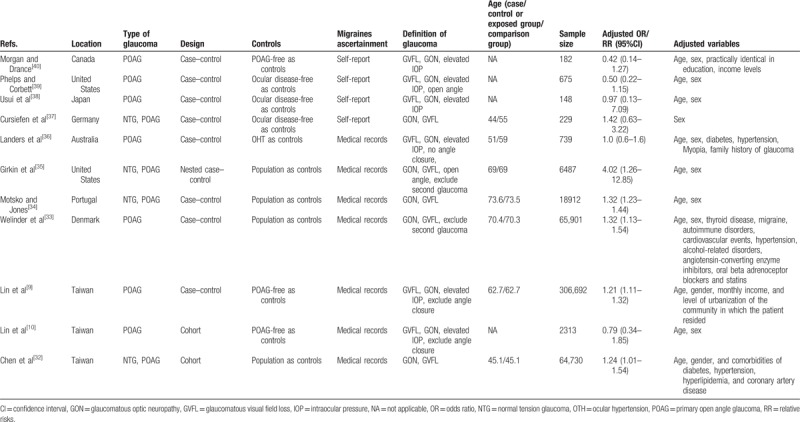
Descriptive characteristics of studies included in the meta-analysis.

**Table 2:**
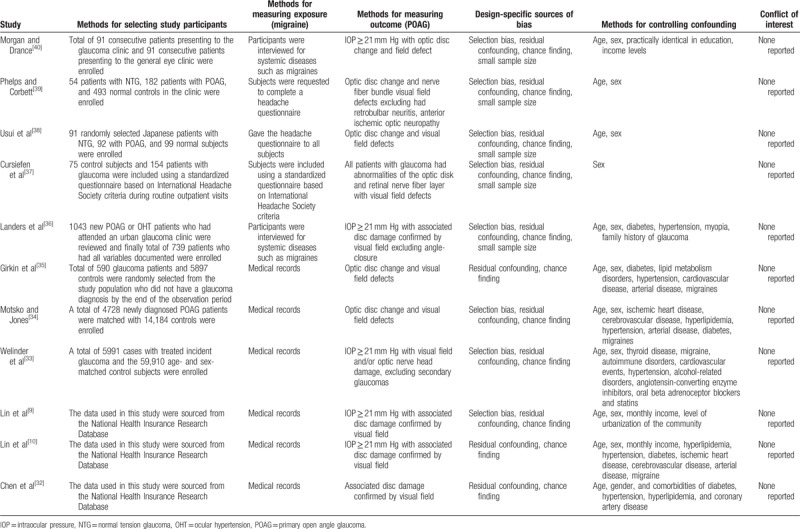
Assessment of methodological quality of included studies on association between migraines and POAG.

### 3.3 Pooled estimates of the association between migraine and POAG

**Figure 2:**
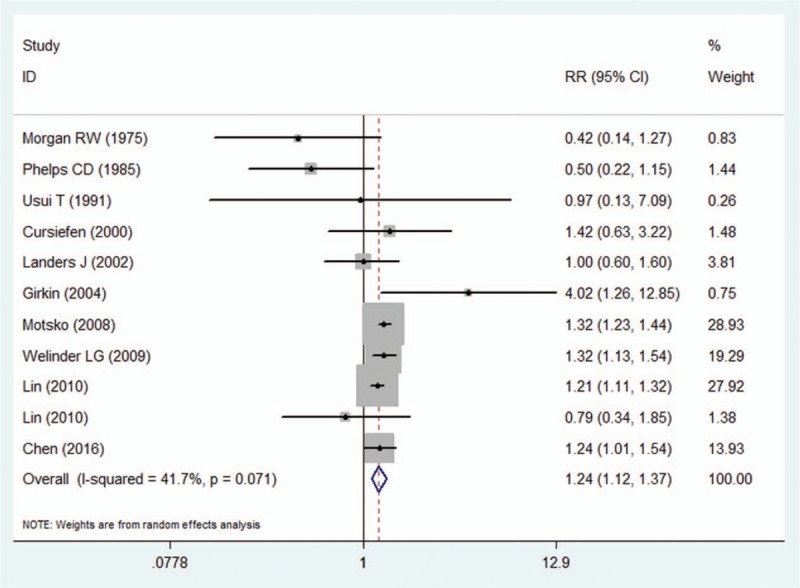
Forest plot of the risk estimates of the association between migraine and POAG.

The pooled effect estimates and the heterogeneity tests of the association between migraine and POAG are presented in Fig. 2. The random-effect model of the 11 included studies indicated a significant association between migraine and increasingly prevalent POAG (RR = 1.24; 95% CI = 1.12–1.37). No evidence of significant heterogeneity was detected across studies (*P* = .071; *I*^2^ = 41.7%). The results of a series of prespecified stratified analyses conducted according to study design, the methods used to determine migraine, geographical area, type of glaucoma, and the adjusted variables are presented in Table 3. In the stratified analysis by study design, the case–control (RR = 1.24; 95% CI = 1.12–1.37) designs demonstrated a significant relationship between migraine and POAG. However, the nested case–control/cohort design (RR = 1.38; 95% CI = 0.72–2.63) did not reveal this association. In the stratified analysis by the methods used to determine migraine, the pooled RR with 95% CI was 1.27 (95% CI = 1.18–1.37) for studies using medical records and 0.73 (95% CI = 0.39–1.37) for studies using self-reports. In terms of subgroup analysis based on geographical area, the relationship between migraine and POAG was more significant for studies conducted in Europe (RR = 1.32; 95% CI = 1.23–1.42) and Asia (RR = 1.21; 95% CI = 1.12–1.31) than in North America (RR = 0.91; 95% CI = 0.24–3.42) and Australia (RR = 1.00; 95% CI = 0.61–1.63). In the included studies, some studies only included POAG patients and others included both POAG and NTG patients, so subgroup analyses were also conducted according to the type of glaucoma. The pooled RR was consistent in the POAG subgroup and the POAG/NTG subgroup and both subgroups showed a significant association between migraine and POAG. The impact of confounding factors on RR was also considered. When the studies were adjusted for age and sex, diabetes, or hypertension, a positive relationship was found between migraines and POAG in all 3 subgroups. No significant heterogeneity was observed in most of the subgroups (Table 4).

**Table 3:**
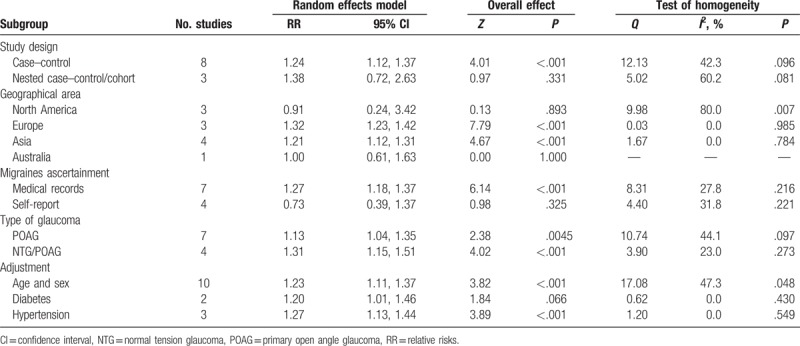
Subgroup meta-analyses of migraine and POAG.

**Table 4:**
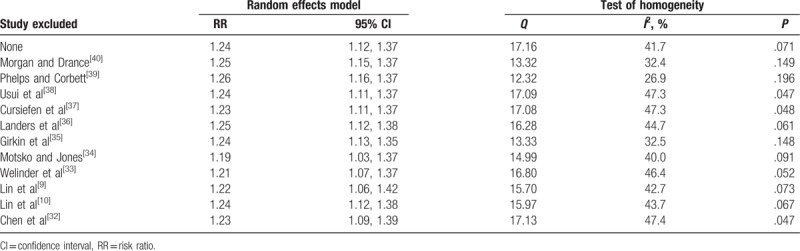
Sensitivity analysis of the included study.

### 3.4 Sensitivity analysis and publication bias

**Figure 3:**
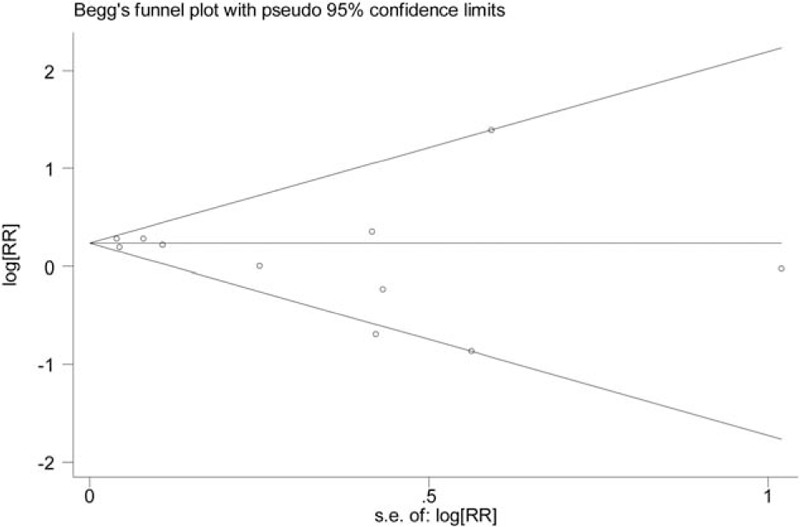
Funnel plot of the included studies evaluating the association between migraine and POAG.

The robustness of the increased risk of POAG incidence due to migraine was evaluated by performing a sensitivity analysis by omitting one study at a time and then calculating the pooled RR for the remaining studies. The results of this “leave-one-out” sensitivity analysis showed that the corresponding global estimation did not change by the deletion of any single study, indicating the robustness of this meta-analysis. We used the Begg funnel plot and Egger test to detect potential publication bias. The value of *P*_Begg test_ and *P*_Egger test_ were .533 and .272, respectively, indicating a low probability of publication bias. The funnel plot for the studies is presented in Fig. 3 and it is symmetrical, which also indicates a low probability of publication bias.

## 4 Discussion

Many risk factors for the development of POAG have been identified,^[3,4,6]^ but the investigation continues. Several publications have reported a correlation between migraine and POAG^[28,32]^; however, no definitive link has yet been established. With this in mind, we conducted this meta-analysis to evaluate this potential relationship. Our examination of the 8 case–control and 3 nested case–control/cohort studies revealed a statistically significant relationship between migraine and POAG. Subjects who suffered from migraine had a 24% higher risk of developing POAG when compared to those who had never suffered from migraine.

The evidence linking migraine and POAG was further strengthened by performing sensitivity and publication bias analyses. Omission of individual studies one at a time and then recalculating the pooled RR for the remaining studies revealed insignificant changes in the corresponding estimates when any single study was deleted, indicating the high stability and reliability of this study. Of note, of the included studies, the study by Landers et al^[36]^ used ocular hypertension subjects as controls, which differed from the other studies that used normal subjects. However, the sensitivity analysis that excluded the Landers et al study^[36]^ also showed no significant change in the pooled RR. Similarly, the publication bias analysis showed a low probability of publication bias, which also implied the robustness of this meta-analysis.

The stratified analyses revealed a more prominent relationship between migraine and POAG in the case–control studies than in the longitudinal studies. Several reasons might explain this difference. First, the small number of included longitudinal studies could have led to an insufficient statistical power to detect a positive association between migraine and POAG in those studies. Second, for longitudinal studies, survival bias may occur, which could mask a real association. Separate analyses of the migraine ascertainment method by medical records and self-report indicated that patients with migraines ascertained by medical records had a 27% increased risk of POAG, whereas the risk of POAG was nonsignificant in those whose migraines were ascertained by self-reports. Self-reporting has its own limitation of recall bias, which might also mask the real association. Among the included studies, some only included POAG patients (IOP > 21 mm Hg), whereas others included both POAG patients (IOP > 21 mm Hg) and NTG patients (IOP ≤ 21 mm Hg). The results suggested that migraine increased the risk of POAG but it also increased the risk of NTG. Notably, the results from the subgroup analyses that were adjusted for age and sex, diabetes, and hypertension, showed a significant association between migraines and POAG. These studies proved to be more reliable than those used in the overall analysis because the true association between migraines and POAG might be diluted by studies that have used poor methodologies.

Our findings suggest an association between migraine and the risk of POAG. However, to date, no mechanisms have been elucidated that could support the notion that migraine could increase the risk of the progression of POAG. One possible explanation might be vascular regulation, as the multifactorial nature of glaucoma is well known and vascular factors play a key role in its pathophysiology, and especially of NTG.^[41]^ The dysregulation of retinal vascular^[23]^ and poor blood flow at the optic nerve head is associated with the incidence of glaucoma.^[42]^ Patients with NTG have reduced blood flow velocities and higher resistive indices in most retrobulbar vessels.^[42]^ Similarly, migraine is also viewed as a vascular disorder to some extent. Charles^[43]^ defined migraine as a disorder with both vascular and neural involvement as part of its pathophysiology. The pain of a migraine is attributed to the activation of the trigeminovascular system.^[44]^ Activation of nociceptors leads to the release of certain vasoactive peptides and inflammatory mediators that act directly to decrease the diameter of cerebral blood vessels.^[44]^ These changes in blood vessel caliber are viewed as indicative of vascular dysregulation or vasospasm. Therefore, potential pathophysiological mechanisms shared in common might form the link between glaucoma and migraine. Some researchers have posited that the relationship between the migraine and POAG is due to a common vasospastic mechanism.^[45]^

Our meta-analysis has several advantages. First, it is the first and the largest analysis, to date, that explores the relationship between migraine and POAG and it represents a comprehensive literature search that included as many relevant studies as possible to provide a more precise conclusion. Second, the sensitivity analysis and the publication bias analysis all confirmed the reliability and robustness of the pooled results. Third, the studies included in this meta-analysis revealed no obvious heterogeneity, indicating a very good homogeneity among the currently available studies. Fourth, study-level data allowed meaningful stratified analyses. This analysis therefore provides the most up-to-date information in the area of the migraine and POAG relationship.

Several limitations of this meta-analysis should also be acknowledged. First, the potential biases in the included studies were not considered. For example, the case–control designs could be subject to selection bias, and the longitudinal studies could be subject to survival bias. Second, in some of the studies, migraine was ascertained by self-reporting, which could have introduced a recall bias. More likely, some migraine patients could be misclassified as nonmigraine subjects. Third, not all the studies controlled for potential confounding variables. However, the results from subgroup analysis restricted to studies adjusted for relative covariates showed a significant association between migraine and POAG, and this is more reliable than the association reported for the overall analysis because the real association might be diluted by studies with poor methodologies. Fourth, some of the subgroup analyses were performed using studies with small numbers and should be interpreted with caution. Fifth, the number of cohort studies included was relatively small and the durations of follow-up of these studies might not be sufficiently long to detect any associations. Finally, in this meta-analysis, the study quality was assessed using the tool described by Sanderson et al.^[12]^ The quality of most of the included studies is relatively high. However, there are still some design-specific sources of bias in most of the studies, and the methods for measuring exposure and outcome is not uniform among the different studies.

In conclusion, the current limited evidence suggests that migraines can significantly increase the risk of developing POAG. However, the cohort study design failed to identify this association. Whether migraines can significantly increase the risk of developing POAG is still controversial. Better designed longitudinal studies with longer follow-ups are required in the future to confirm the association between migraines and POAG.

## Author contributions

**Conceptualization:** Chang Xu.

**Data curation:** Jingjing Li.

**Formal analysis:** Jingjing Li.

**Methodology:** Zhi Li.

**Project administration:** Chang Xu.

**Resources:** Zhi Li.

**Supervision:** Xiaochun Mao.

**Writing – original draft:** Chang Xu.

**Writing – review & editing:** Xiaochun Mao.
